# The sustainability of lean implementations at the hospitals of Ministry of Health Malaysia: A study protocol

**DOI:** 10.1371/journal.pone.0294055

**Published:** 2023-11-13

**Authors:** Kah Yee Lum, Ku Anis Shazura binti Indera Putera, Muniamal Krishnan, Zalina binti Libasin, Nur Nadia Renu binti Abdullah, Intan Syafinaz binti Saman@Saimy

**Affiliations:** Institute for Health Management, National Institutes of Health, Ministry of Health Malaysia, Setia Alam, Malaysia; Higher Education Partnership / Erasmus University Rotterdam, ETHIOPIA

## Abstract

**Introduction:**

In Malaysia, lean was initiated in 2012 as part of an effort to reduce waiting time at the Ministry of Health (MOH) hospitals. As of now, there are 52 public hospitals that have officially implemented lean. However, little is known whether lean is sustained within the hospitals and the critical success factors (CSFs) affecting sustainability. Therefore, this study protocol aims to fill the gap by (i) identifying the critical success factors [CSFs] for lean sustainability in the MOH, (ii) developing a validated framework to support hospitals in sustaining lean, (iii) the framework will be adapted into a checklist to measure the level of lean sustainability (iv) interviewing hospitals with the lowest and highest level of lean sustainability to further explore the barriers and boosters in sustaining lean.

**Methods and analysis:**

This study will employ a mixed-method approach and will be conducted in three phases. The first phase involves a combination of scoping review and interviews with key informants to identify the CSFs known to affect lean sustainability at the MOH hospitals and present them in a validated framework. In the second phase, the framework will be adapted into a checklist to measure the level of lean sustainability in the MOH hospitals. The findings will be used to select the hospital with the lowest and highest level of lean sustainability for an interview in the third phase.

**Discussion:**

The lean sustainability framework will be able to provide more relevant guidance on how to increase the likelihood of lean sustainability and serve as a validated measurement tool for MOH hospitals. In addition, this study will be able to outline the differences in the contributing factors between health organizations that showed a high level of lean sustainability compared to those struggling to sustain.

## Introduction

With health expenditures expected to rise with advances in medical technologies, ageing, and rising public expectations, there is a growing interest in healthcare to implement lean. Lean concepts were initially developed for use in manufacturing, and it is unknown when lean was adapted to the healthcare sector. The first publications on lean healthcare were dated from 2002. Since then, lean has been extensively implemented worldwide owing to its ability to focus on both customer satisfaction and employee involvement [1].

In Malaysia, lean was initiated in 2012 to reduce waiting time at the Ministry of Health [MOH] hospitals [2]. The pilot hospitals chosen for implementation showed positive improvements in waiting time, admission capacities, and overcrowding [3]. Since lean has been proven to be an efficient process improvement, the MOH further expanded lean to 52 public hospitals in an agile approach [2, 4]. The agile approach consists of a six to twelve-month program that includes training on lean awareness with on-site consultation and monitoring. However, these hospitals have demonstrated difficulties in achieving the ideal sustained state of lean [3]. Furthermore, monitoring the progress and maintenance of lean in the MOH are based on performance indicators that could only assess the strategic intent of Lean without indicating if lean is sustainable [3, 5].

Similarly, studies in other parts of the world have reported the lack of lean sustainability in health organizations, citing lean improvements tend to revert to their original state over time [6]. Initiatives that fail to be sustained are extremely wasteful of resources [7]. To benefit the most from the substantial investment in lean, organizations need to better understand the factors that can encourage long-term program sustainability [8]. The sustainability of lean relies on various critical success factors (CSFs) such as employees’ roles, behaviour and engagement, work characteristics, and leadership [9]. These CSFs are hypothesized to be precursors of lean sustainability [10].

A previously developed framework by Henrique et al. [11] has proposed 22 main CSFs that can be used to assess the sustainability of lean in healthcare. However, the framework could not be directly adopted by the MOH as the hospitals recruited in that study were comprised of private healthcare systems. The contextual differences in the public and private healthcare of lean implementations, particularly in their differing views on patient-centred values, can influence the factors attributed to lean sustainability in the organizations [12]. As the hospitals in MOH operate on a public healthcare system, there is a need to develop a framework adapted to our local health settings that can be utilized by MOH Malaysia.

Therefore, this study protocol aims to (i) identify the CSFs for lean sustainability in the MOH and use it to (ii) develop a validated framework to support hospitals in sustaining lean and (iii) adapt the framework to a measurement tool in the form of checklist to measure the level of lean sustainability and lastly (iv) conduct a qualitative interview among hospitals with the lowest and highest level of lean sustainability to further explore the barriers and boosters in sustaining lean.

## Methods

**Fig 1:**
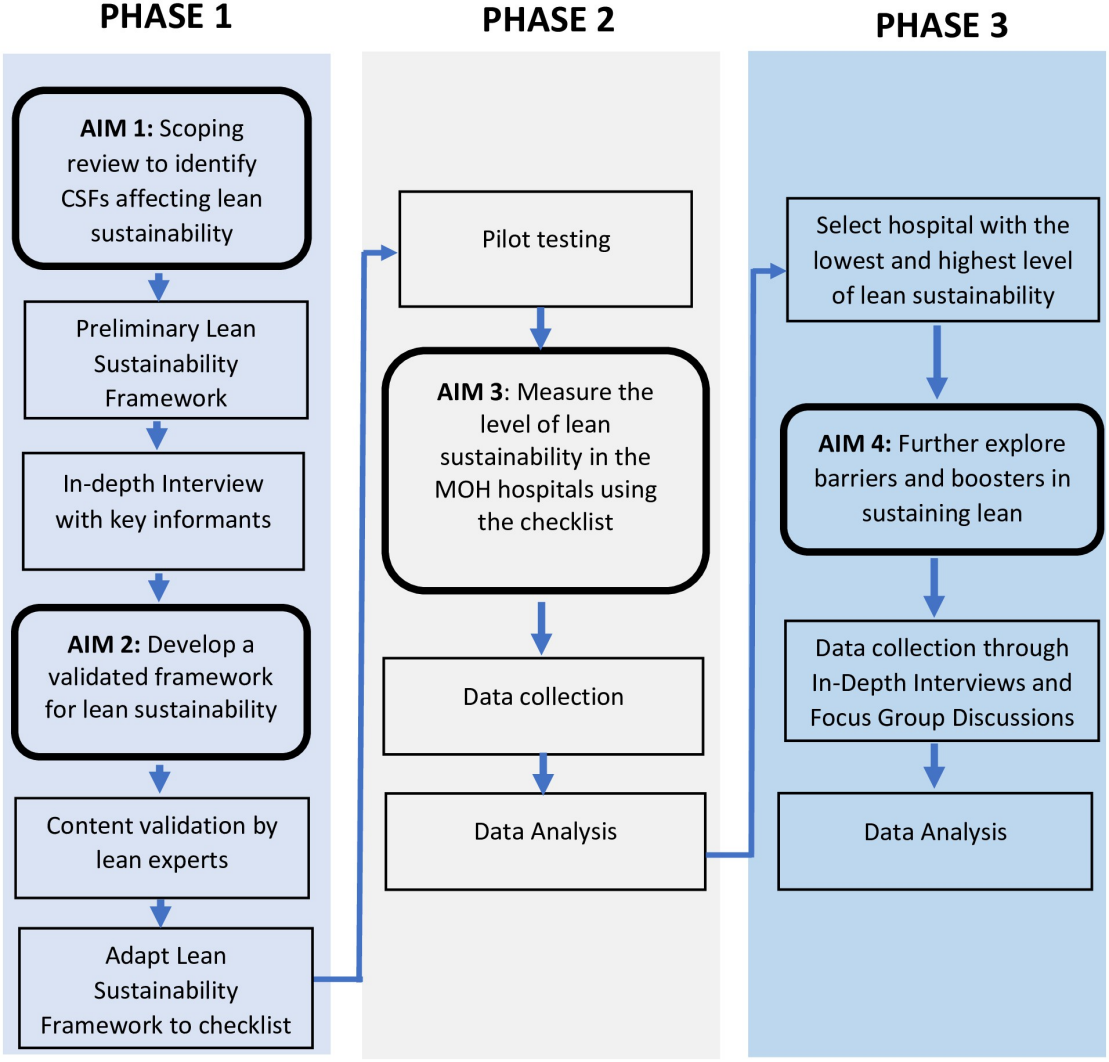
Study workflow.

This study will employ a mixed-method approach consisting of qualitative and quantitative components, as research on sustainability typically consists of a mix of surveys, audits, and interviews to assess potential influences on sustainability [13]. It will be conducted in three phases, as summarized in Fig 1. The first phase involves a combination of scoping review and qualitative interviews with key informants to identify the CSFs known to affect lean sustainability at MOH hospitals, and the findings will be presented in a validated framework. The second phase will be quantitative, whereby the checklist derived from the framework will be utilized to measure the level of lean sustainability in the MOH hospitals. Subsequently, the results from the second phase will be used to select the hospital with the lowest and highest level of lean sustainability for a further qualitative interview in the third phase.

### Phase 1: Framework development that includes scoping review and interviews with key informants to identify the CSFs affecting lean sustainability in the MOH hospitals

#### (a) Scoping review

This scoping review will be guided by the Preferred Reporting Items for Systematic Reviews and Meta-Analyses extension for Scoping Reviews (PRISMA-ScR) [14]. The searches will be formulated using the PIC (Population, Intervention, and Context) framework with Medline, PubMed, Emerald Insight, and Google Scholar as the search engines. Citation searching will be done to identify additional relevant articles from the bibliographies of the retrieved articles. The following terms and synonyms will be used in the search engines and combined to produce final search strings such as (health OR healthcare OR hospital OR “primary care”) AND (lean OR “process improvement”) AND (sustainability OR sustain* OR "critical success factor" OR maintenance).

The articles must be related to lean healthcare to be eligible for inclusion. Secondly, all study designs will be considered including qualitative and quantitative studies. Finally, articles must be reported in English and published between 2012 to 2022. Articles will be excluded if the context is non-health such as lean in manufacturing and ongoing studies, protocol, and book chapters. Two reviewers will independently screen the (i) title and abstracts of articles identified from the initial search and (ii) full-text articles will be retrieved and reviewed according to the pre-specified inclusion and exclusion criteria. Ambiguity about whether a study should be included or excluded will be resolved through a third reviewer.

A standardized extraction form will be used to extract general information about the study, its design, and information on the CSFs. Data will be extracted in duplicates, stored, and managed using Google Forms and Microsoft Excel. The research team will determine the most suitable method of analysis once the data has been collected. It is anticipated that a thematic analysis will be used to explore the relationships among the identified data.

#### (b) Qualitative interview with key informants

From the scoping review, a preliminary sustainability framework consisting of CSFs affecting lean sustainability in healthcare will be developed. It will be further refined using a qualitative approach to ensure relevancy and to identify new factors that can contribute to the sustainment of lean before a final framework is established.

For each site, the researchers will identify a site liaison officer (LO) to assist in coordinating and identifying suitable participants for interviews. The interview participants will be purposively sampled to include policymakers who are responsible for the initiation and monitoring of lean implementations in the MOH hospitals as well as healthcare workers (HCWs) in the top and mid-managerial positions (e.g. hospital director, chief of departments and middle managers) working in an accredited lean hospital. An In-Depth Interview (IDI) approach will be used. The interview guide will be based on the framework derived from the findings of the scoping review. This study will tailor the interview according to the participant’s occupational positions. The interview questions will be used to explore the perspectives of policymakers related to lean sustainability such as resource allocation, monitoring, and integration of lean culture. On the other hand, questions for HCWs will elicit their views on how motivations, employee involvement, and leadership affect lean sustainability. Supporting documents such as internal audit reports of lean performance indicators, projects, value stream maps, A3 reports, training, and presentations will be collected to minimize retrospective bias. Data saturation will be considered once there are no new emerging themes from the last interview.

The interviews will be audio-recorded and transcribed using N-vivo software. The data will be coded in a deductive approach based on the preliminary sustainability framework developed during the scoping review. New themes emerging from the interviews will be added to the existing framework.

#### (c) Framework validation

The purpose of this framework development is to propose a checklist for assessing the level of lean sustainability in hospitals. Therefore, validation is essential to ensure the adequacy of the framework. According to Inglis 2008 [15], there is a lack of a standardized approach for framework validation and proposed the use of expert panels as one of the main validation methods. A consultative discussion with lean experts consisting of 10 participants from a multi-disciplinary background will be conducted. The panel size is characterized by the experience and knowledge of the panel rather than the number. Therefore, the researchers deemed 10 experts will be sufficient for the validation. The experts will be asked to comment on the relevance of the key domains and to provide any suggestions for improvement in a feedback form.

Next, the framework will be adapted to a measurement tool for lean sustainability in the form of a checklist that comprises all the identified CSFs. The design of the checklist and scoring methods will also be discussed with the experts. The research team will then review the findings and make necessary amendments before pilot testing.

### Phase 2: Pilot testing of the checklist and measurement on the level of lean sustainability

#### (a) Pilot testing

The checklist will be pilot-tested at one of the public hospitals that have been involved in the first phase of lean-agile. The LO at the study site will help to identify HCWs in the top, mid-managerial positions and front liners as participants in the pilot study. Using the checklist, the participants will be interviewed in a close-ended approach to assess the presence of the CSFs in the hospital. This would allow the researchers to estimate the time and resources needed to collect the data in the checklist as well as to ascertain the reliability. As the checklist will be administered by the researchers, testing for inter-rater reliability is essential to ensure the data collected accurately represents the variables under observation. It will be obtained using Cohen’s kappa index with an index above 0.60 to indicate adequate agreement [16].

#### (b) Quantitative measurement on the level of lean sustainability

The checklist will then be used to measure the level of lean sustainability in the MOH hospitals. The findings from this phase would be able to indicate whether lean is sustaining and at what level in the MOH hospitals. Six hospitals will be randomly sampled from the hospitals involved in the first phase of lean agile (excluding the hospital involved in pilot testing). The six hospitals will be representative of the six regions in Malaysia. This is based on stakeholders’ expert opinion as it is presumed that these hospitals should be suitable to be assessed for lean sustainability.

The data will be collected through interviews with HCWs in the top and mid-managerial positions and supplemented with reviews of documents. By engaging the LOs, this study will recruit at minimum one officer in top management positions and one officer in a mid-managerial role from each study site as the researchers postulate that only this group will be able to answer the checklist and sufficiently represent the study site.

Based on the developed checklist, the participants will be interviewed using a closed-ended approach on the existence of each of the CSFs. Each of the CSFs will be scored according to the suggestions by expert panels as elaborated in Phase 1 Section C. Their responses will be verified with relevant supporting evidence. Triangulation of data will be performed through examination of official documents that could indicate if lean is sustaining such internal audit and A3 reports and kaizen presentations. At the end of the interview, feedback from the interviewers will be provided to verify the observations.

The scores from the checklist will be summed to provide the overall level of lean sustainability for each hospital. Based on the total score, the hospitals be grouped into a high, medium, and low level of lean sustainability. This will assist in the identification of differences between the groups to provide a better understanding of the barriers and boosters in sustaining lean which will be explored in phase 3.

### Phase 3: Qualitative interviews in hospitals with the lowest and highest level of lean sustainability

The hospital with the lowest and highest level of lean sustainability identified in phase 2 will be recruited for the interview. The interview will further explore the barriers and boosters in sustaining lean in their hospitals. Purposive sampling will be applied for the selection of HCWs to be interviewed. The samples will represent all categories of work positions (i.e. hospital directors, managers, chiefs of departments, supervisors, front-liners) and professional backgrounds (i.e. physicians, nurses, paramedics, pharmacists). It is vital to cover a multitude of different professional backgrounds as sustaining lean requires the direct involvement of staff from all organizational levels.

A semi-structured guide interview will be constructed based on the Consolidated Framework for Implementation Research (CFIR). This study finds CFIR suitable to be used as a guiding framework as it can consider a broad scope of factors related to sustainability and it can be used to identify potential barriers and boosters [17]. The interview questions will be related to the five domains in CFIR: intervention characteristics, outer setting, inner setting, characteristics of individuals, and process.

The study team will engage two LOs, one from each of the study sites. The LOs will assist in identifying participants with suitable experience in lean implementations. A different interview approach will be utilized depending on the positions of the HCWs: IDIs for HCWs in the top and mid managerial positions while focus group discussion (FGD) would be more suited for front liners. A study by Hennink and Kaiser [18] suggested a range of 9 to 17 as a sample size for IDIs and 4 to 8 focus groups for FGDs. However, this number may differ in this study based on data saturation, where no new information can be obtained. The interview session will be audio-recorded and a note-taker will be present to record relevant non-verbal communication. Data collection will continue until saturation.

The interview will be transcribed verbatim and coded independently by two researchers, according to the domains in the CFIR framework. An inductive coding approach will be used to explore emerging themes that are not covered in the CFIR. The team members will then compare the coding, agree on the main constructs and subconstructs and discuss any disagreements. The analysis will be done using NVivo to facilitate data management and coding.

## The status and timeline of the study

This study has commenced data collection in June 2023 and is expected to end by June 2024.

## Discussion

Interest in sustainability is a growing field as policymakers are concerned about whether their interventions continue after an initial investment, especially in health systems with finite resources. It is critical to understand the organizational or contextual elements that affect the sustainment of a particular program. It is also important to examine every aspect of sustainability at the level of organizations, teams, and individuals and establish methods for determining if the intervention effects are indeed sustained [19]. However, much of the evidence on lean sustainability remains theoretical, offering minimal guidance on how to sustain program delivery, implementation strategies, and outcomes [13, 20]. Therefore, this research can contribute to the limited evidence by increasing the understanding of what factors are important in the sustainability of lean in healthcare. It is important to address these factors preferably at the beginning of lean implementation to achieve the sustainment of lean. The findings from this study will also benefit policymakers and lean practitioners involved in evidence-based lean implementations in the MOH. First, the developed lean sustainability framework will be tailored to our local contexts as it will be tested by its application in selected public hospitals. This will provide more relevant and applicable guidance on how to increase the likelihood of lean sustainability in MOH hospitals. Furthermore, the framework will be validated by a multidisciplinary team of experts to reduce the level of bias thereby enhancing the robustness of the framework. On the other hand, as the validation largely relied on the experts’ knowledge and may not constitute the best-informed answer, the framework will be tested and applied in real-world settings as outlined in Phase 2 of the study.

Second, this study will be able to outline the differences in the contributing factors between health organizations that showed a high level of lean sustainability compared to those struggling to sustain. This will assist in mitigating contextual barriers and strengthening facilitators to allow for more efficient planning towards lean sustainability. The empirical data generated from the study would allow policymakers to develop and select implementation strategies to enhance lean sustainability that is grounded on study findings.

There will be some limitations to our study. The proposed framework in Phase 1 will be derived from in-depth interviews with respondents from hospital settings. Thus the framework might not be generalisable for primary care facilities with different health systems and resources. Moreover, the sample size of six randomly selected hospitals from Phase 2, which were part of the initial phase of lean implementations in the MOH, may not be adequate for conducting quantitative theory testing using statistical methods.

In conclusion, it is expected that this study will shed some light on the determinants of lean sustainability and the degree of lean sustainability among the MOH hospitals in Malaysia. The results from this study would also allow other ASEAN countries with similar health contexts to compare and contrast the contributing determinants in lean sustainability.
